# Measuring Dissociation Rate Constants of Protein Complexes through Subunit Exchange: Experimental Design and Theoretical Modeling

**DOI:** 10.1371/journal.pone.0028827

**Published:** 2011-12-14

**Authors:** Chongle Pan

**Affiliations:** Oak Ridge National Laboratory, Computer Science and Mathematics Division and BioSciences Division, Oak Ridge, Tennessee, United States of America; Russian Academy of Sciences, Institute for Biological Instrumentation, Russian Federation

## Abstract

Protein complexes are dynamic macromolecules that constantly dissociate into, and simultaneously are assembled from, free subunits. Dissociation rate constants, *k_off_*, provide structural and functional information on protein complexes. However, because all existing methods for measuring *k_off_* require high-quality purification and specific modifications of protein complexes, dissociation kinetics has only been studied for a small set of model complexes. Here, we propose a new method, called Metabolically-labeled Affinity-tagged Subunit Exchange (MASE), to measure *k_off_* using metabolic stable isotope labeling, affinity purification and mass spectrometry. MASE is based on a subunit exchange process between an unlabeled affinity-tagged variant and a metabolically-labeled untagged variant of a complex. The subunit exchange process was modeled theoretically for a heterodimeric complex. The results showed that *k_off_* determines, and hence can be estimated from, the observed rate of subunit exchange. This study provided the theoretical foundation for future experiments that can validate and apply the MASE method.

## Introduction

Many biological processes are carried out by protein complexes that are assembled from multiple subunits. As subunits are generally bound together by non-covalent interactions, protein complexes can reversibly dissociate into free subunits. Assembly and disassembly of complexes are reversible processes that can reach dynamic equilibrium. The kinetics of the two processes is characterized by the association rate constant (*k_on_*) and the dissociation rate constant (*k_off_*). The association/dissociation reaction of a simple heterodimeric complex *AB* comprising a subunit *A* and a subunit *B* can be represented as:
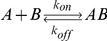
Because diffusion is often the key rate-limiting factor for protein association, *k_on_* for protein complexes is generally in the order of magnitude of 10^6^ s^−1^ [1]. On the other hand, *k_off_* is dictated by the strength of short-range interactions between subunits, such as van der Waals interactions, hydrogen bonds, hydrophobic interactions and ionic bonds [2]. As a result, different complexes have a wide range of *k_off_* and environmental condition changes can also significantly alter a complex's *k_off_*. Study of protein interaction kinetics has provided valuable insights into protein complexes and their functions [1], [3].

The main existing methods for measuring *k_off_* of a complex are surface plasmon resonance (SPR) and the stopped-flow method [4]. An SPR analysis starts by immobilizing purified intact complexes onto the SPR sensor surface. *k_off_* is measured by monitoring the dissociation of the complex through the change of the refractive index near the SPR sensor surface. The stopped-flow method relies on the presence of a fluorescence probe in the complex, which enables detection of protein interaction using fluorescence signal. Except for a few complexes with intrinsic fluorescence probes, most complexes need to be modified to introduce an extrinsic fluorescence probe. It takes a large amount of effort to purify and prepare a complex such that it can be analyzed using the two methods. As a result, to our best knowledge, so far there are less than 20 complexes with known *k_off_*, other than antigen-antibody systems [3], [4], [5].

If the intact assembly of a complex can be ionized with minimum disruption to its quaternary structure, mass spectrometry can used to measure the complex directly [6], [7]. This enabled characterization of composition, subunit stoichiometry, conform change, and assembly of complexes [8]. If every subunit of a complex can be isolated and the complex can be assembled from the purified free subunits, a pulse-chase quantitative mass spectrometry method (PC/QMS) method can be used to determine the association rate constant, *k_on_*, of the complex [9]. This method was used to study the self-assembly process of the 30S subunit of the bacterial ribosome [9].

Here a new method, called Metabolically-labeled Affinity-tagged Subunit Exchange (MASE), is proposed for measuring *k_off_* of protein complexes using two general high-throughput techniques: affinity purification and quantitative proteomics. In a tandem affinity purification (TAP) experiment, an affinity tag is inserted into one of the subunits of a complex by genetic engineering [10]. The tagged subunit is isolated using two sequential rounds of affinity purification. Because gentle purification conditions are used, the intact complex is purified along with the target protein. Subunits of the complex are identified with a shotgun proteomics analysis. TAP is a high-throughput and generic technique for identification of subunits in protein complexes. Using this approach, thousands of protein complexes have been characterized in yeast [11], [12] and *E. coli* [13]. Quantitative proteomics allows accurate quantification of relative abundances of proteins using stable isotopic labeling and mass spectrometry. The chemical isotopic labeling method, Isotope-Coded Affinity Tags or ICAT, was used to distinguish genuine subunits of a complex from co-purifying contaminants in a TAP analysis and to measure the composition change of a complex in different conditions [14]. Metabolic labeling was used in an I-DIRT (Isotopic Differentiation of Interactions as Random or Targeted) approach to distinguish bona fide subunits of a complex from contaminants in a TAP analysis [15]. The wide applicability and high throughput of TAP and quantitative proteomics were demonstrated in these studies.

In the MASE method, an unlabeled affinity-tagged variant and a ^15^N-labeled untagged variant of a complex are allowed to exchange their constituent subunits in a crude cell lysate mixture for varying periods of time. The progress of subunit exchange can be measured using affinity purification and quantitative proteomics. In this study, theoretical modeling showed that the rate of subunit exchange is determined by and only by *k_off_*. Thus, *k_off_* of a complex can be estimated by measuring its subunit exchange rate in a MASE experiment. We believe that this study shows the potential of the new method, paving the way for experimental approaches that will be of interest to biologists with a need for measuring *k_off_* of protein complexes.

## Analysis

### Experimental design of the subunit exchange method

**Figure 1:**
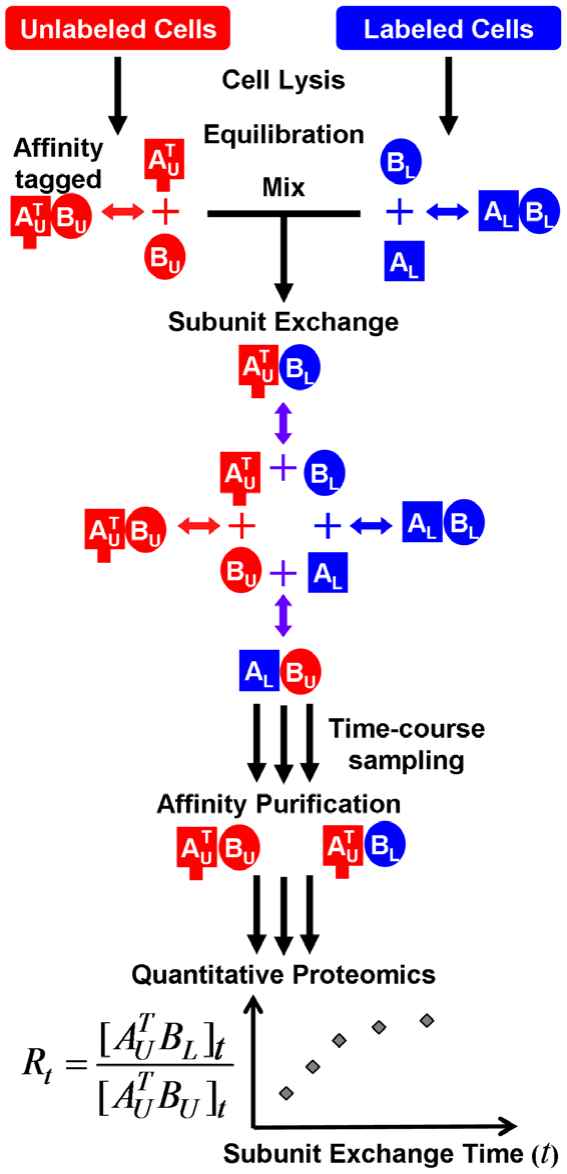
Overview of the MASE method. The system for a heterodimeric complex, *AB*, consists of an affinity-tagged unlabeled subunit *A* (*A^T^_U_*, red square), an unlabeled subunit *B* (*B_U_*, red circle), a labeled subunit *A* (*A_L_*, blue square), and a labeled subunit *B* (*B_L_*, blue circle). The subunit exchange process produces hybrid complexes, *A^T^_U_B_L_* and *A_L_B_U_*, until reaching equilibrium. Abundance ratios (*R_t_*) between the two tagged complexes, *A^T^_U_B_L_* and *A^T^_U_B_U_*, at different time points of subunit exchange are determined by affinity purification and quantitative proteomics. A time series of *R_t_* can be used to estimate *k_off_*.

The proposed MASE method is illustrated in Figure 1 using a heterodimeric complex *AB*. A recombinant strain of a microorganism is constructed to express an affinity-tagged subunit *A*. The affinity tag allows affinity purification of complex *AB*. Because subunits of the complex are known and co-purifying contaminants would not interfere with the analysis, a simple affinity tag can be used for single-stage affinity purification. Homologous recombination should be used such that subunit *A* is expressed only in the affinity-tagged form in the recombinant strain at the same abundance level as in the wildtype strain. Pilot experiments may need to be performed to check that recombination, protein expression, and complex isolation can be achieved as expected. An unlabeled culture of the recombinant strain is grown in a normal growth medium to express unlabeled tagged complex *AB*. A metabolically labeled culture of the wildtype strain is grown in a ^15^N- or ^13^C-labeled growth medium to express labeled untagged complex *AB*. It is expected that the two variants of this complex in the two cultures are biologically equivalent. For clarity, let *A^T^_U_* and *B_U_* denote unlabeled affinity-tagged subunit *A* and unlabeled subunit *B*, respectively, in the unlabeled recombinant culture. Let *A_L_* and *B_L_* be labeled subunit *A* and labeled subunit *B*, respectively, in the metabolically labeled wildtype culture.

A MASE experiment involves three steps: subunit exchange, affinity purification, and quantitative proteomics analysis (Fig. 1). In the first step, the same number of unlabeled recombinant cells and labeled wildtype cells are lysed separately in the same volume of a subunit exchange buffer. The two cell lysates are incubated for an extended period of time to equilibrate the associate/dissociation reaction of the complex in the subunit exchange buffer. The subunit exchange buffer contains a full-spectrum cocktail of protease inhibitors to suppress protein degradation. The two cell lysates are then mixed and further incubated to allow for subunit exchange. The equilibration and subunit exchange should occur under a condition of interest for studying *k_off_*, including appropriate temperature, pH, ionic strength, co-factor concentrations, *etc*. Initially, all complexes are either *A^T^_U_B_U_* from the unlabeled cells or *A_L_B_L_* from the labeled cells. As time passes, complexes continuously dissociate into free subunits and are re-assembled from free subunits (Fig. 1). Consider *A^T^_U_* in this process. *A^T^_U_* continuously dissociates from *B_U_* at the beginning and re-associates with free B, which can be *B_U_* or *B_L_*. Association of free *A^T^_U_* and free *B_L_* generates hybrid tagged complex *A^T^_U_B_L_*, whose concentration increases with time until reaching equilibrium with the original tagged complex *A^T^_U_B_U_*. This process can be viewed as if *B_U_* is replaced by *B_L_* gradually in the tagged complex until reaching equilibrium. The progress of subunit exchange is represented by the increase of [*A^T^_U_B_L_*] relative to [*A^T^_U_B_U_*]. Let us define 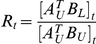 at time point *t*. To measure the increase of *R_t_* with time, aliquots of the subunit exchange sample are retrieved in different time points.

In the second step, the tagged complexes, *A^T^_U_B_L_* and *A^T^_U_B_U_*, in every sample collected at different time points are isolated using affinity purification. Briefly, samples are incubated with affinity capture beads. The complexes containing the affinity tag, *A^T^_U_B_L_* and *A^T^_U_B_U_*, and the tagged free subunit *A^T^_U_* are immobilized on the affinity capture beads; whereas the untagged complexes, *A_U_B_L_* and *A_U_B_U_*, and other proteins are in the solution. The beads are then washed to remove unbound proteins, including free subunits *A_L_*, *B_L_* and *B_U_* and complexes *A_L_B_U_* and *A_L_B_L_*. Finally, *A^T^_U_B_L_*, *A^T^_U_B_U_* and *A^T^_U_* are eluted off from the beads. We expect that *R_t_* remains the same after affinity purification, because the two isotopic variants, *A^T^_U_B_L_* and *A^T^_U_B_U_*, should have identical purification efficiency.

In the third step, the isolated complex is measured using a quantitative proteomics approach. Briefly, samples are digested using trypsin and analyzed using liquid chromatography coupled with tandem mass spectrometry (LC-MS/MS) [16]. Because of the low sample complexity, only one-dimensional reverse-phase liquid chromatography (RP-LC) is needed. Peptides eluted off the RP-LC column are ionized by electrospray and analyzed using tandem mass spectrometry. Proteins are identified from tandem mass spectra using a database searching algorithm. Abundance ratios between unlabeled and labeled variants of proteins are then determined from selected ion chromatograms of their constituent peptides [17]. Because affinity purification samples should be enriched in *A^T^_U_B_L_*, *A^T^_U_B_U_* and *A^T^_U_* and have no free *B_L_* or *B_U_*, the quantitative proteomics measurement provides the abundance ratio between bound *B_L_* and bound *B_U_* in *A^T^_U_B_L_* and *A^T^_U_B_U_*, *i.e. R_t_*. The time series of *R_t_* measured from all collected samples represents the progress of subunit exchange.

### Theoretical modeling of the subunit exchange process

Subunit exchange can be modeled mathematically by considering thermodynamics and kinetics of the association/dissociation reactions involved in this process. Let us first consider the reactions involving *A^T^_U_*:
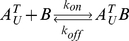
(1)where subunit *B* can be either *B_L_* or *B_U_* because the two isotopic variants, *B_L_* and *B_U_*, are biologically equivalent. Before mixing, reaction (1) is equilibrated in the unlabeled cell lysate:
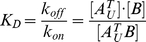
(2)where [*A^T^_U_*], [*B*], and [*A^T^_U_B*] are constant with time. After the unlabeled cell lysate is mixed with the same volume of the labeled cell lysate, [*A^T^_U_*] and [*A^T^_U_B*] are diluted by a factor of two, but [*B*] is not changed with the addition of the same quantity of *B_L_* from the labeled cell lysate. Because the dilution of [*A^T^_U_*] and [*A^T^_U_B*] cancels out in equation (2), the mixing does not disrupt the equilibrium of reaction (1). Therefore, from the beginning through the entire time course of subunit exchange, reaction (1) remains at equilibrium and [*A^T^_U_*], [*B*], and [*A^T^_U_B*] are maintained constant. The same logic can be applied to the reactions involving *B_U_*, *A_L_* and *B_L_*. Hence, the following two propositions hold:

Proposition (1): the concentrations of free subunits, [*A^T^_U_*], [*B_U_*], [*A_L_*] and [*B_L_*], are constant during subunit exchange.

Proposition (2): the concentration of 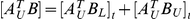 is constant during subunit exchange, where [*A^T^_U_B_L_*]*_t_* and [*A^T^_U_B_U_*]*_t_* are the concentrations of *A^T^_U_B_L_* and *A^T^_U_B_U_*, respectively, at arbitrary time point *t*.

Next, consider the two reactions involving *A^T^_U_* separately:
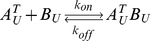
(3)

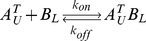
(4)At the beginning of the subunit exchange, reaction (3) moves in the direction of dissociation; whereas reaction (4) moves in the direction of association. At *t* = ∞ when both reactions reach equilibrium,
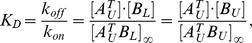
(5)where [*B_U_*] = [*B_L_*] based on Proposition (1). Thus, we can derive 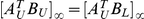. Based on Proposition (2), 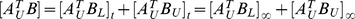. Hence, Proposition (3) holds:

Proposition (3): During subunit exchange, *B_U_* is replaced by *B_L_* in *A^T^_U_B* until an equilibrium is reached, in which 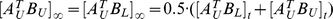.

Finally, consider reaction (4) that produces *A^T^_U_B_L_*. The kinetics of reaction (4) can be described as:
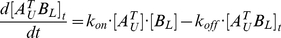
(6)From equation (6), we can deduce:
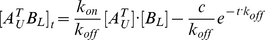
(7)where *c* is a constant (See Proof S1 for a step-by-step deduction). At equilibrium *t* = ∞,
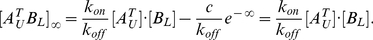
(8)Combine equation (7) and equation (8):
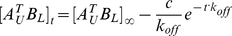
(9)Because *A^T^_U_B_L_* does not exist at the beginning of the subunit exchange,
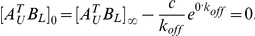
Therefore, 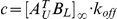 and substitute *c* in equation (9):
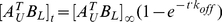
(10)Substitute 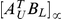 in equation (10) using Proposition (3):
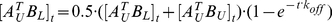
(11)Rearrange equation (11) to derive *R_t_*:
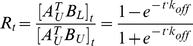
(12)Equation (12) shows that the value of *R_t_* at time point *t* is determined by and only by *k_off_*.

**Figure 2:**
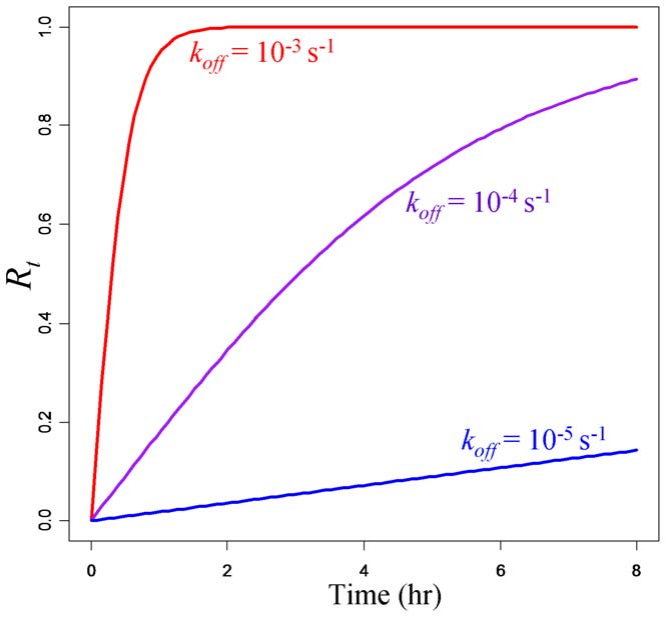
Theoretical modeling of subunit exchange at different *k_off_* values. Subunit exchange progress measured by a time series of *R_t_* is determined by *k_off_* as defined in equation (12). Time series of *R_t_* are calculated for the first 8 hours of subunit exchange using *k_off_* at 10^−3^ s^−1^, 10^−4^ s^−1^ and 10^−5^ s^−1^. Higher *k_off_* results in faster subunit exchange.

Theoretical exchange progress curves were calculated for the first 8 hours at different orders of magnitude of *k_off_* (Figure 2). The subunit exchange reached the equilibrium at *R_t_* = 1 in two hours for *k_off_* = 10^−3^ s^−1^, whereas the exchange progressed only to *R_t_* = 0.14 in eight hours for *k_off_* = 10^−5^ s^−1^. As described in the previous section, a time series of *R_t_* is measured in a subunit exchange experiment. Thus, a complex's *k_off_* can be inferred by fitting an exchange progress curve to the measured values of *R_t_* at different time points. Least-square fitting can be obtained readily using a numerical approach.

## Discussion

In comparison to the existing methods for *k_off_* analysis, the most significant advantage of the MASE method is that it might be readily applied to a large number of protein complexes. SPR can only be used to measure complexes that can be highly purified and effectively immobilized, which may require a large amount of experimental effort to accomplish for a complex of interest. The stopped-flow method can only be applied to complexes with fluorescence signal that can be switched on and off by protein interactions. It is not trivial to engineer such an extrinsic fluorescence probe into a complex. In comparison, MASE only requires homologous expression of an affinity-tagged subunit of a complex in a model microorganism. Thousands of protein complexes have been isolated by affinity purification from yeast [11], [12] and *E. coli* [13] in previous large-scale interactomics studies. The MASE method uses mass spectrometry to monitor the exchange process of isotopically labeled subunits in a complex. The sensitivity and specificity of mass spectrometry obviates the need for preparation of large quantity of highly purified complexes or their subunits. Thus, we believe that the MASE method can be an attractive alternative method for future dissociation kinetics studies.

This study only examined the subunit exchange process of a simple heterodimeric complex. The MASE method could also shed light on the disassembly of multi-subunit complexes. By tagging one subunit in a complex, one can build a subunit exchange progress curve for each of the remaining subunits. An apparent *k_off_* can be derived for each subunit's dissociation from the tagged subunit by assuming the other subunits have no effect on the interaction of those two subunits. However, it will be challenging to infer the true disassembly kinetics of the complex from the apparent *k_off_* of individual subunits. Complications could arise from a sequential disassembly process of a complex and cooperative assembly/disassembly among subunits. Note that the existing methods are also affected by these complications of multi-subunit complexes.

In this study we described an experimental design of the MASE method. Our theoretical work showed that the subunit exchange process of a heterodimeric complex can be used to estimate its *k_off_*. We believe this method is experimentally feasible, and should provide a new tool for biophysical characterization of protein complexes. Because of the complexity of the proposed subunit exchange experiment, a significant amount of work will be needed to optimize the methodology and establish it as a valid approach for *k_off_* measurement.

## Supporting Information

Proof S1 **Step-by-step deduction of** **equation (7** **).**
(DOC)
