# Supplementary material for: Measuring Dissociation Rate Constants of Protein Complexes through Subunit Exchange: Experimental Design and Theoretical Modeling
Source: PLoS One. 2011 Dec 14;6(12):e28827. doi: 10.1371/journal.pone.0028827 (PMC3237551; doi:10.1371/journal.pone.0028827)
Supplement: Proof S1 — Step-by-step deduction of equation (7 ). (DOC) [file pone.0028827.s001.doc]

**Supplemental Proof S1:**

Subunit exchange involves the following association/dissociation reactions:

To derive a generic formula, let represent any one of the above association/dissociation reactions. Based on Proposition (1), [*A*] and [*B*] are constants. *k_off_* and *k_on_* are also constants.

At equilibrium *t* = ∞, $[AB]_{\infty}=\frac{k_{on}}{k_{off}}[A]\cdot[B]-\frac{c}{k_{off}}e^{-\infty}=\frac{k_{on}}{k_{off}}[A]\cdot[B]$.

Therefore,

Note that the constant, *c*, will have different values in different association/dissociation reactions. In the main text, the constant *c* in is specific to the reaction .
